# Non-Volatile Compounds Involved in Bitterness and Astringency of Pulses: A Review

**DOI:** 10.3390/molecules28083298

**Published:** 2023-04-07

**Authors:** Adeline Karolkowski, Christine Belloir, Loïc Briand, Christian Salles

**Affiliations:** 1 Centre des Sciences du Goût et de l’Alimentation, CNRS, INRAE, Institut Agro, Université de Bourgogne, F-21000 Dijon, France; adeline.karolkowski@inrae.fr (A.K.); christine.belloir@inrae.fr (C.B.); 2 Groupe Soufflet (Ets J. Soufflet), 10400 Nogent-sur-Seine, France

**Keywords:** pulses, off-flavours, bitterness, astringency, TAS2R, saponins, phenolic compounds, alkaloids

## Abstract

Despite the many advantages of pulses, they are characterised by off-flavours that limit their consumption. Off-notes, bitterness and astringency contribute to negative perceptions of pulses. Several hypotheses have assumed that non-volatile compounds, including saponins, phenolic compounds, and alkaloids, are responsible for pulse bitterness and astringency. This review aims to provide an overview highlighting the non-volatile compounds identified in pulses and their bitter and/or astringent characteristics to suggest their potential involvement in pulse off-flavours. Sensorial analyses are mainly used to describe the bitterness and astringency of molecules. However, in vitro cellular assays have shown the activation of bitter taste receptors by many phenolic compounds, suggesting their potential involvement in pulse bitterness. A better knowledge of the non-volatile compounds involved in the off-flavours should enable the creation of efficient strategies to limit their impact on overall perception and increase consumer acceptability.

## 1. Introduction

Pulses, also called dried legume seeds, present nutritional, environmental, and functional interests compared to animal proteins [1,2,3]. However, the presence of off-flavours in these legumes constitutes an important obstacle to their consumption, which limits their use in food applications [4].

Negative organoleptic perceptions are named off-flavours or unpleasant flavours. They are the combination of off-notes (unpleasant odours and aromas), off-tastes (unpleasant tastes), and unpleasant trigeminal sensations. The off-notes in pulses are described as beany, grassy, pea, rancid, metallic, etc. and are due to the presence of some volatile compounds originating from seed metabolism during farming and process conditions and are now well-identified in pulses [4,5]. In contrast, much less is known about the taste compounds of pulses. Taste perceptions are caused by compounds that activate receptors on the tongue and the oral cavity [6]. Among the five basic tastes, bitterness is the only one to have been identified as an off-taste in pulses [4]. Another negative perception, astringency, which is a native trigeminal sensation, has been inventoried in many legumes, such as beans, lentils, and peas [7]. However, little is known about non-volatile compounds that are responsible for these off-flavours in pulses. These compounds are the object of this review.

Some molecules, including saponins, phenolic compounds, alkaloids, peptides, and free amino acids, are involved in the bitterness and/or astringency of pulses [4,8]. Moreover, new research suggests that 14 lipids and lipid oxidation products are involved in the bitterness of pea protein isolates [9]. Despite their potential involvement in pulse off-flavours, it is important to note that saponins, phenolic compounds, and alkaloids (detected in lupins and faba beans) come from a secondary metabolism and contribute to plant defences in the case of biotic and abiotic stresses [10,11,12,13,14]. For example, the content of saponins was greater in pea samples exposed to very intense contamination with pathogens [11]. Research has mainly focused on the overall bitterness and/or astringency of pulse-based products and correlated these intensities with the compound content [15,16]. However, the number of potential non-volatile compounds is so important that it remains complicated to demonstrate their direct involvement in these off-flavours. Some researchers have extracted potential compounds from pulses of interest and have studied their bitter and/or astringent characteristics using sensorial analyses. For example, the extract of soyasaponins βb and DDMP (2,3-dihydro-2,5-dihydroxy-6-methyl-4H-pyran-4-one) in peas was described as bitter and astringent by panellists, which suggested their role in pea off-flavours [17]. Sensorial analyses are also completed via in vitro assays that highlight the interaction between a molecule and human bitter taste receptors (TAS2R—human type 2 taste receptor). Genistein and daidzein, two isoflavones in soybeans, were identified as bitter by panellists [18,19] and activated the TAS2R14 and TAS2R39 receptors [20,21]. These molecules should have an important contribution to bitterness in soybeans. Although the role of some compounds in the bitterness and astringency of pulses has been identified, there are still many hypotheses to be confirmed.

This review is focused on pulses. It aims to propose a concise overview highlighting the non-volatile compounds potentially responsible for their off-flavours. The first part defines the unpleasant perceptions in pulses related to non-nasal detection, such as bitterness and astringency, and compares in vitro and in vivo methods for the identification of bitter and/or astringent compounds. The second part highlights the non-volatile compounds (saponins, phenolic compounds and alkaloids) detected in pulses that have also been identified as bitter and/or astringent using sensorial analysis in legumes or other food products and through cellular-based functional TAS2R receptor assay. This cross-referencing of information would make it possible to identify new compounds responsible for off-flavours in pulses. Although peptides and free amino acids may be involved in pulse off-flavours, their sensorial aspect will not be discussed in this review because they are mainly formed during protein hydrolysis (fermentation/enzymatic hydrolysis, heat treatment, and wet protein extraction) [22,23]. Moreover, the tastes of free amino acids including their bitter activity have already been reviewed [24].

## 2. Highlighting of Bitter Taste and Astringent Perception

This part aims to define the bitter taste and astringency, a trigeminal sensation, that contribute to off-flavours in pulses. The bitter and astringent characteristics of molecules are often determined through sensorial analysis (in vivo methods). In addition, in vitro cellular assays have been used to measure receptor activation by sapid compounds and thus determine their involvement in bitterness. These in vivo and in vitro approaches are compared in this review.

### 2.1. Generalities on Bitterness and Astringency

- Bitterness

**Figure 1:**
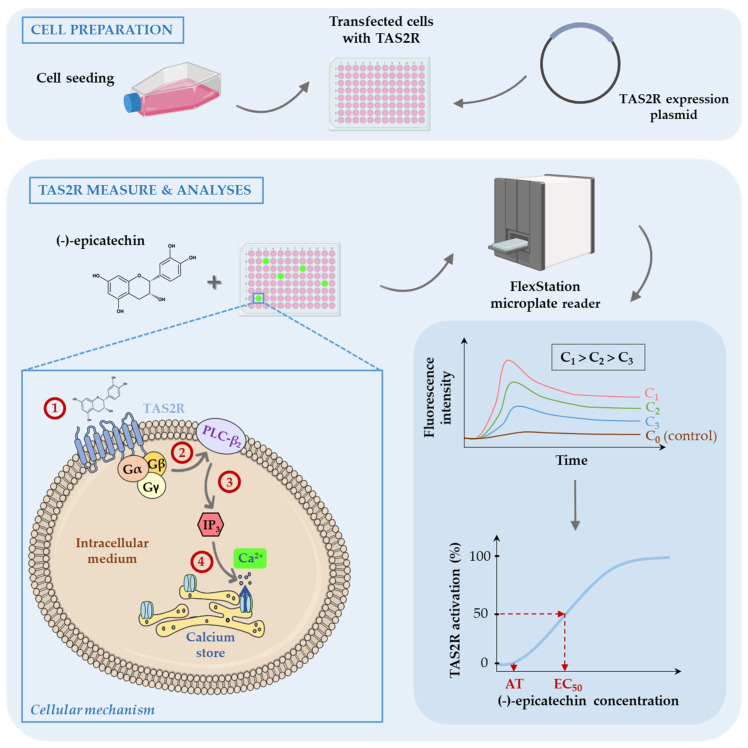
In vitro cellular test method used to study the activation of TAS2R by non-volatile compounds (for example, (-)-epicatechin) (adapted from [28,29]). Cellular mechanism—The activation of the receptor results in the dissociation of the chimeric Gα16Gust44 subunit (Gα-gustducin) from the βγ-subunits (Step ①). The βγ-subunits activate phospholipase C-β2 (PLC-β2) (Step ②), resulting in the formation of the second messenger inositol 1,4,5-triphosphate (IP3) (Step ③) [30]. Then, IP3 binds to its endoplasmic reticulum receptor, leading to the transient release of Ca^2+^ (from calcium stores) that is detected using a calcium-sensitive dye (Step ④) [31]. Most of the icons come from BioRender. TAS2R: type 2 taste receptor; C: concentration; AT: activation threshold; EC_50_: half-maximum effective concentration to achieve 50% of the maximum amplitude of receptor activation.

Bitterness is often unappreciated and an indicator of potential toxicity and/or bacterial contamination of foods to limit their ingestion [25]. The detection of bitter molecules, with a wide range of chemical structures, is achieved by a family of G protein-coupled receptors (GPRC) called TAS2Rs [6,26]. GPCRs share a common architecture with seven transmembrane domains and a signal transduction mechanism involving a heterotrimeric G protein [27]. The transduction pathway is briefly presented in Figure 1 (see cellular mechanism).

Humans have ~25 putative functional TAS2Rs [32]. The perception of bitter compounds varies widely between individuals due to genetic variations linked to polymorphism [33,34,35,36,37,38,39]. For example, individuals with the TAS2R38-AVI (alanine–valine–isoleucine) variant are not able to detect bitterness in a solution of phenylthiocarbamide, whereas individuals with the TAS2R38-PAV (proline–alanine–valine) polymorphism are able to detect this compound [40,41]. Many factors such as age, pathology, medication, dietary habits and salivary composition, have been shown to alter bitter perception [30,31,42].

- Astringency

Astringency is one of the trigeminal sensations. Astringent molecules interact with the hydrophobic “pocket” of salivary proteins and lead to their precipitation. This causes drying, roughening, and puckering of the mouth epithelia. Tannins and other phenolic compounds are well-known to be astringent molecules. The polymer size, concentration, and galloylation of tannins can modify the astringent intensity [6]. However, the mechanism of astringency is still not as well-understood as that of bitterness. This perception may be detected by mechanoreceptors in the oral mucosa after an increase in friction forces at the surface of epithelial cells [43,44] or through the detection of the aggregation of the mucosa pellicle by transmembrane mucin (MUC1) [45]. Tannins exhibit a higher astringency threshold than flavonols. They are a source of harsh, drying and puckering astringent mouthfeels whereas flavonols induce velvety and silky mouthfeels [46,47]. These differences may be explained by two phenomena. The interactions between salivary and proteins may be more related to harsh, dry and puckering sensations, while interactions driven by the tongue or buccal mucosa cell lines may be more related to velvety and silky sensations [48]. These hypotheses still need to be tested with sensory results.

### 2.2. Sensory Identification of Bitterness and Astringency (In Vivo Test)

Sensory analyses have been used to describe the taste of pure compounds, especially bitterness and astringency. They reflect an overall sensation of bitterness/astringency perceived by a panel of individuals and consider the integration of the signal at the cerebral level [49]. First, panellists are trained to be familiar with the studied perceptions. In most studies, panellists are required to wear a nose-clip to prevent olfactory perceptions from impacting bitterness and astringency. Second, the human bitter or astringent detection threshold (DT) is determined with the three-alternative forced choice test consisting of a triangle test with two blank samples and ascending concentrations for each compound. For each concentration, panellists must identify the solution that exhibits bitterness or astringency among the three samples. The DT is obtained by calculating the geometric mean of the last missed concentration and the next higher concentration detected [40,50,51,52,53,54]. Third, panellists must evaluate the bitter or astringent intensity of different concentrations of the studied compound. Dose–response curves are constructed by plotting the bitter or astringent intensity as a function of the molecule concentration. The human EC_50_ corresponds to the concentration of molecule required to achieve 50% bitter or astringent intensity [40,52,53].

Many studies have focused on the overall bitterness and/or astringency of legumes and legume-based products. Sometimes, the sensory properties are correlated to chemical analysis to predict the molecules responsible for the perceptions of interest [15,55,56,57]. For example, the correlation of phytochemical compositions and sensory attributes of pea protein fractions has predicted the bitterness and astringency of 29 compounds [15]. Then, these results can be validated using sensory and/or cellular approaches to pure compounds; however, these are rarely carried out for economic reasons or are not permitted for food applications.

### 2.3. Activation of the Human Bitter Taste Receptors TAS2Rs (In Vitro)

In vitro cell-based assays allow for the measuring of the activation of bitter taste receptors under physiological conditions similar to their environment using sapid compounds [28,58]. This approach has the advantage of studying synthetic or toxic molecules that are not part of the human diet, unlike sensory analysis [52].

The in vitro assay is usually performed with HEK293 cells stably expressing a chimeric Gα-protein (Gα16Gust44), as presented in Figure 1. The cells were seeded in a microplate and transfected with a plasmid encoding for the studied TAS2R. The cells produce a functional TAS2R embedded in the plasma membrane. The studied molecule is injected into the wells at different concentrations, and TAS2R activation causes a signalling cascade resulting in the release of calcium from the endoplasmic reticulum into the intracellular medium. Then, cytoplasmic calcium binds a fluorescent indicator (Fluo-4 or Fura-2) [51] or a genetically encoded calcium biosensor (GCaMP) [59,60]. More recently, a bioluminescence-based assay was developed for TAS2R to measure calcium mobilization using mt-clytin II, a calcium-sensitive photoprotein, and the luminophore coelenterazine [61]. The calcium signal is measured through fluorescence or luminescence using a fluorometric imaging microplate reader (FlexStation or FLIPR). The response of TAS2R depends on the concentration of bitter compounds and leads to different signal amplitudes [28,58,61]. Then, the AT (activation threshold) is determined, corresponding to the lowest concentration for which fluorescence is observed. Dose–response curves are constructed by plotting the signal amplitude as a function of the molecule concentration. The EC_50_, which corresponds to the concentration of agonist required to achieve 50% of the maximum amplitude of receptor activation, is also determined and can be compared to relative bitterness in vivo [28]. The use of cellular-based assays with functional expression of TAS2Rs in human HEK293 cells showed that some bitter taste receptors have a large broad detection spectrum (TAS2R14), while others were intermediate (TAS2R4, TAS2R7, TAS2R30, TAS2R39 and TAS2R43) or were activated by a few molecules (TAS2R5). Some molecules activate several TAS2Rs, such as (-)-epicatechin with TAS2R4, TAS2R5, TAS2R14 and TAS2R39 [20,62,63,64]. However, no agonist has yet been identified for TAS2R19, TAS2R42, TAS2R45 and TAS2R60 [58,65].

### 2.4. Correlation between Sensorial and In Vitro Analyses of Bitterness

Some studies have correlated in vivo and in vitro results to bitterness (Table 1) [40,51,52,53]. However, the link between cellular data and human analyses is not always effective. Indeed, the DT and human EC_50_ are often greater than the AT and the in vitro EC_50_, respectively. For example, the ratio of DT/AT was 11 times greater for vitamin B1 [51] and 566.7 times greater for cohumulone [52]. These observations can be explained as based on different phenomena. In vitro cellular-based assays are performed in buffer media with a composition that differs from that of human saliva. This can decrease the availability of compounds for the receptors due to their complexation with hydrophobic proline-rich proteins in the saliva and/or be adsorbed by the oral epithelium [52,66]. Hydrophobic bitter compounds are also more sequestered by oral proteins and/or mucosa than polar compounds, which can increase the difference between human and cellular results [52]. Delompré et al. (2022) [51] proposed that sample dilution via saliva can also be responsible for an increase in the human bitter taste DT [51]. Moreover, flavour perception is a cerebral construction resulting from the integration of chemosensory signals in the brain arising from the ingestion of an ingredient. There are several steps in brain information treatment provided by neural signals, including external signals that can interact with taste perception [49,51]. Although the in vitro method enables the rapid identification of bitter compounds and activated TAS2Rs, it is not suitable for all compounds. For example, fluorescence related to calcium release was detected in non-transfected cells (absence of TAS2Rs) upon the addition of high concentrations of vitamins B2 and B3, whereas sensory analyses revealed the bitterness of these compounds [51]. The in vitro cellular results should thus always be verified through sensory analyses on pure compounds when possible.

## 3. Non-Volatile Compounds Involved in Bitterness and Astringency of Pulses

This section is devoted to non-volatile compounds (including saponins, phenolic compounds and alkaloids), which have been both detected in pulses and identified as bitter and/or astringent through sensorial and/or in vitro analysis. Sensory analyses are not limited to pulses but extend to all food products. This approach makes it possible to summarise the compounds identified as bitter and/or astringent in legumes and to propose non-volatile compounds that could be involved in these off-flavours.

Soybeans are less studied in this review due to their high oil concentration compared to other pulses, including adzuki beans, beans, chickpeas, faba beans, lentils, peas and lupins. However, some soybean examples have been addressed when the literature was sparse concerning other pulses, especially for saponins and isoflavones.

### 3.1. Saponins

**Figure 2:**
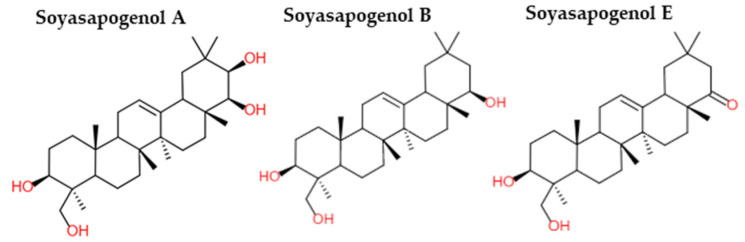
Chemical structures of soyasapogenol A, B and E (adapted from [73]).

Saponins are amphiphile molecules. They consist of a steroidal or triterpene hydrophobic aglycone and one to three sugars (hydrophilic part) attached by ester or ether linkage [68]. In legumes, triterpenoid saponins were the main saponins identified, although some steroidal saponins were also detected [69,70,71]. In soybeans, saponins were found in cotyledons and derived from soyasapogenols A, B, and E (Figure 2) [68]. The amount of saponins in legume seeds was very different according to their type. Chickpeas contained 2.6 to 60 g/kg (dry matter) of saponins against 0.1 to 3.7 g/kg (dry matter) for broad beans and 1.8 to 11 g/kg (dry matter) for green peas [72]. 

**Figure 3:**
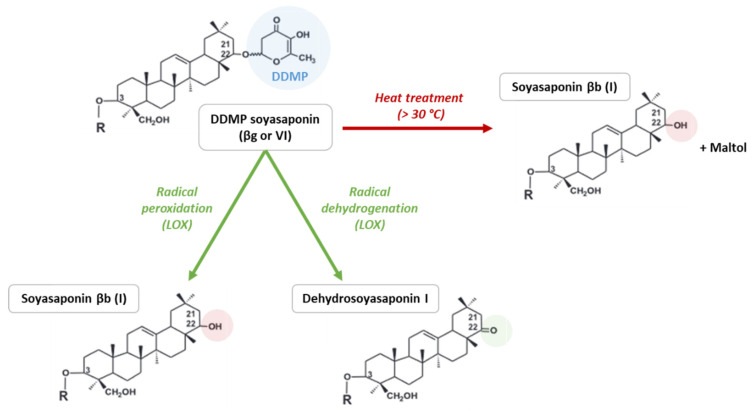
Degradation of DDMP soyasaponin into soyasaponin βb and dehydrosoyasaponin I through heat treatment and enzymatic reactions (adapted from [78]). LOX: lipoxygenase; R: glucuronic acid-galactose-rhamnose.

In peas, an extract of isolated soyasaponin βb (soyasaponin I) is described as bitter and astringent. Moreover, the protein fraction obtained through air-classification should contain sufficient saponins to detect these off-flavours [74]. Indeed, saponins interact with proteins and are found more easily in the protein fraction than in the starch fraction [75,76]. Concerning another pea study, soyasaponin βb and DDMP soyasaponin (also called soyasaponin βg, soyasaponin VI or chromosaponin I) were extracted and sensorially evaluated by thirteen trained panellists. The bitterness DT of soyasaponin βb is approximately 8 mg/L in water, and a mixture of soyasaponin βb and DDMP soyasaponin in a ratio of 1:4 is less than 2 mg/L in water [17]. Indeed, DDMP soyasaponin is degraded into soyasaponin βb + maltol at temperatures greater than 30 °C, which makes it difficult to extract and purify [17,77,78,79]. Maltol has a caramel-like odour and a sweet taste that can modify the flavour. Moreover, DDMP soyasaponin is also degraded by lipoxygenase during grinding due to an important amount of dioxygen. The peroxidation of DDMP soyasaponin leads to soyasaponin βb, whereas dehydrogenation leads to dehydrosoyasaponin I [17,78]. The degradation process is presented in Figure 3. The effect of soyasaponin βb on the bitterness and astringency of pea protein isolate has been reported by calculating dose–over-threshold factors (the ratio of compound concentration over bitter/astringency threshold for each compound). The factor for astringency is 1.8 and 0.7 for bitterness, suggesting that soyasaponin I is more involved in astringency than in bitter taste [9]. However, DDMP soyasaponin has not been detected and has probably been degraded due to high-temperature extraction, which can reduce the bitter intensity. In another study, the areas of the main phytochemical compounds identified through ultrahigh-performance liquid chromatography–diode array detector–mass spectrometry (UHPLC-DAD-MS) of pea-based samples were correlated with sensory attributes to model bitterness and astringency (prediction). According to the modelling, saponin B, saponin derivates, and soyasapogenol B are not involved in bitterness, whereas they contribute to astringency [15]. However, the DDMP soyasaponin has a priori not been detected in these samples (absence of a peak with a mass of approximatively 1067 g/mol [70,71]), probably due to the high-temperature extraction used (40 °C) that degrades it into soyasaponin βb and/or soyasaponin E [78]. In soybeans, different saponin fractions have been extracted and sensorially characterised. Soyasaponins A, B, and E have bitter characteristics and exhibit 10^−5^, 5.10^−4^ and 10^−3^ mM taste threshold values, respectively. Aglycones A and E are slightly bitter and present a lower threshold value. An astringent perception has been identified in soyasaponin A and soyasapogenol B [80]. Soybean extracts with different soyasaponin βb concentrations exhibit the same off-flavour intensities. The DDMP saponin (and malonyl-β-glucoside isoflavones) should contribute more to bitterness and astringency than the other saponins and isoflavones identified in these extracts [81]. Although the sensory contribution of saponins has only been studied in soybeans and peas, it is important to note that DDMP soyasaponin and soyasaponin βb have been identified in other pulses including adzuki beans, common beans, lentils, chickpeas, lupins and broad beans [70,71,82]. However, the level of saponins in lupin seeds is very low, suggesting no significant contribution to bitterness and astringency [16].

Researchers have produced saponin-free pea varieties (the study was focused on DDMP and βb soyasaponins) [77]; however, it would have been interesting to compare the bitter and astringent intensities between wild and mutant cultivars to verify the effect of saponin content on sensory properties. A possible drawback of such an approach is that both saponins and volatile compounds interact with proteins; consequently, the decrease in saponin content may increase protein-volatile compound interactions and impact the odour/aroma of pulses [83]. The 10-day germination of broad beans increases the saponin content [71] and may intensify bitter and astringent perceptions. Finally, the heating of pulses appears to be a simple and efficient strategy to reduce these off-flavours in pulses due to the degradation of the DDMP soyasaponin [78,84,85].

### 3.2. Phenolic Compounds

Phenolic compounds are a large class of plant secondary metabolites exhibiting a diversity of structures. They have one or more hydroxyl groups attached directly to the aromatic ring and vary from simple molecules to highly complex polymers [86]. In pulses, many groups have been identified, including phenolic acids (and their derivates), stilbenes, flavonoids, and condensed tannins [87]. Their content in legumes depends on the cultivar, location (including abiotic and biotic stress conditions), and transformation [87,88,89,90,91]. They protect plant tissues against UV (ultraviolet) irradiation and participate in plant defence against herbivores, fungi, and viruses [87]. White-flowered faba beans are associated with low tannin due to breeding. These low-tannin genotypes have been reported to be more susceptible to soil-borne diseases [89]. Moreover, phenolic compounds are distributed differently in the parts of the seed (Table 2). The hulls of chickpeas, faba beans, and lentils contain higher amounts of tannins, whereas the cotyledons are richer in other phenolic compounds, including flavonoids [90,91]. During food processing and storage, plant phenolic compounds are converted to a variety of reaction products that modify the product flavour [87].

Phenolic compounds are mostly studied for their beneficial effects on human health rather than their sensorial characteristics. However, they are responsible for bitterness and astringency in legumes [4]. The astringent intensity of phenolic compound extracts of different pulses has been evaluated by a trained panel that classified them in the following order: red beans > adzuki beans > lentils > peas > broad beans > faba beans [7]. Moreover, studies on the activation of human bitter taste receptors by many phenolic compounds suggest that they are involved in pulse off-flavours [20,21,62,66]. The concentration and bitter/astringent characteristics of phenolic compounds identified in pulses are presented in Table 3. It is important to note that extraction methods are different and comparisons are complex.

#### 3.2.1. Phenolic Acids and Derivates

Many phenolic acids and their derivates have been identified in pulses (Table 3). Phenolic acids contribute to bitterness but more significantly to astringency in wine or corn germ protein flour [50,92,93]. Beans, lentils, and peas exhibit a low concentration of p-hydroxybenzoic acid (0.32–1.0 µg/g), whereas peas are richer in protocatechuic acid (2.06–221 µg/g) and lentils in p-coumaric acid (3.22–3.42 µg/g) [94]. m-Coumaric acid has been detected in faba beans and peas [71,95]. Chickpeas and lentils contain a high amount of gallic acid, and chickpeas are richer in caffeic acid than other pulses [90,96,97]. These six phenolic acids identified in pulses are well-known to be responsible for astringency in wine. Protocatechuic, p-coumaric, m-coumaric, gallic, and caffeic acids exhibit moderate bitterness at a concentration of 2 g/L in water, whereas p-hydroxybenzoic acid is described as being slightly more bitter [92]. These phenolic acids could contribute to pulse bitterness.

According to the correlation model, caffeic acid contributes to both bitterness and astringency in peas, with an estimated concentration of 90.7 ng/g [15].

#### 3.2.2. Stilbenes

Two stilbenes have been identified in faba beans, resveratrol and polydatin [98]. Resveratrol is often produced by plants as a defence against microbial infections, upon exposure to UV and other stresses [99,100]. Bitterness and astringency have only been studied for resveratrol, which is known to contribute to the bitterness and astringency of wine [101]. This molecule is described as bitter at a concentration of 47 mg/L in water [67]. Moreover, cellular tests have shown that resveratrol activates the bitter receptors TAS2R14 and TAS2R39. TAS2R14 is more sensitive to this compound than TAS2R39 [20]. It would have been interesting to quantify resveratrol in faba beans [98] and to compare this concentration with the cellular test results [20] to determine its involvement in bitterness.

#### 3.2.3. Flavonoids

**Figure 4:**
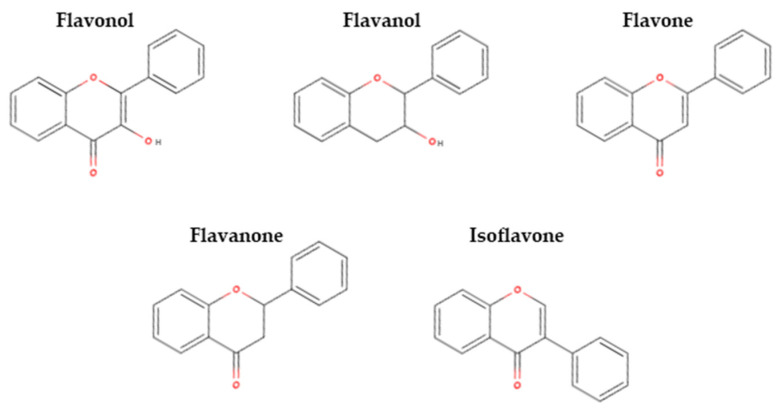
Chemical structures of the flavonoids (adapted from [87]).

Flavonoids are the largest group of phenolic compounds. Some flavonols, flavanols, flavones, flavanones, and isoflavones are identified as astringent and/or bitter (sensory and in vitro tests), which may imply their potential impact on pulse off-flavours. The structures of these flavonoids are presented in Figure 4.

- Flavonols

Flavonols have a 3-hydroxyflavone backbone according to the place and number of OH groups. Kaempferol, quercetin and myricetin activate TAS2R14 and TAS2R39. Kaempferol exhibits lower AT (8 µM for TAS2R14 and 0.5 µM for TAS2R39) than myricetin (250 µM for TAS2R14 and 0.5 µM for TAS2R39), while quercetin leads to ambiguous activation at 500 µM [20]. Chickpeas contain a high content of these three flavonols (5.5–97.5 µg of kaempferol/g dry matter (DM)), but the variation in concentration depends on genotype, location, and seed sample (whole, dehulled, seed coat, and embryonic axe) [90,96]. Adzuki beans are also rich in quercetin (36.2 µg/g) [102]. In addition, many derivates from these three flavonols have also been identified in legumes [15,86,103,104,105]. In particular, quercetin-3-O-glucoside, quantified in pea flour at a concentration of 14.8 ng/g, was found to contribute to pea flour astringency according to the correlation model [15].

- Flavanols

Flavanols are isomers of (+)-catechin and/or (+)-gallocatechin, and participate in the formation of condensed tannins. Several flavanols have been detected in pulses, some of which have been characterised as bitter and astringent, such as (+)-catechin, (-)-epicatechin, (-)-epigallocatechin, (-)-epicatechin gallate, (-)-epigallocatechin gallate and theaflavin [66,92,106,107]. Beans and chickpeas exhibit higher concentrations of (+)-catechin, whereas faba beans have high concentrations of (-)-epicatechin gallate and (-)-epigallocatechin gallate [96,97,108,109,110] (Table 3).

These six flavanols identified in pulses are described as astringent but exhibit different DTs between 16 and 930 µmol/L. Theaflavin, only detected in faba beans, has a very low DT (16 µmol/L) compared to (+)-catechin (410 µmol/L) and (-)-epicatechin (930 µmol/L) [107]. Their sensory description suggests their involvement in the astringent characteristics of pulses.

The activation of bitter receptors by these flavanols has been studied (Table 4). The receptor TAS2R39 is activated by all the flavanols identified in pulses. According to different studies, the AT and EC_50_ are different for (-)-epicatechin, (-)-epicatechin gallate, and (-)-epigallocatechin gallate [20,62,63,64,66]. Theaflavin exhibits a very low EC_50_ (2.79 µM) compared to (-)-epicatechin gallate (21.3 µM) and (-)-epigallocatechin gallate (112 µM) [64]. The same ranking was observed in another study but with a higher EC_50_, 88.2 µM for (-)-epicatechin gallate and 181.6 µM for (-)-epigallocatechin gallate [63]. Moreover, (-)-epigallocatechin and (-)-epicatechin exhibit higher EC_50_ values for TAS2R39 [62,63]. Concerning TAS2R14, (-)-epicatechin and theaflavin have not activated this receptor, in contrast to TAS2R39 [64]. Flavonoids are made of two to three OH groups, which should be involved in hydrogen bonds with TAS2R39. Moreover, the receptor binding pocket of TAS2R39 exhibits an additional acceptor site compared to TAS2R14, which could explain its high affinity [20]. (-)-Epigallocatechin gallate exhibits a higher TAS2R4 and TAS2R5 AT than (-)-epicatechin [62,66]. Finally, (-)-epigallocatechin gallate activates both TAS2R30 and TAS2R43 [66]. This flavanol should be more involved in pulse off-flavours due to the activation of many receptors and low AT compared to the other compounds. Further studies on a wider range of flavanols should allow a better overview of bitter taste receptor activation by these compounds.

**Table 3:** Phenolic compounds identified in pulses and their bitter and astringent characteristics.

Phenolic Compounds	CAS *	M * (g/mol)	Pulses						Bitterness		Astringency
			Adzuki Beans	Beans **	Chickpeas	Faba Beans	Lentils	Peas	Sensory Evaluation	TAS2R Evaluation	
**PHENOLIC ACIDS**											
p-Hydroxybenzoic acid	99-96-7	138.1		0.32–0.36 µg/g [94] 5.05 µg/g DM [103] 10.33 µg/g DM [104] 4.03–12.20 µg/g [108]	2.1–44.4 µg/g DM [90] 1.6–56.6 µg/g DM [96]	4.7 µg/g [111] 0.44–1.11 µg/g [88]	0.94–1.00 µg/g DM [94] 3.75 µg/g DM [103] 73.46 µg/g [112] 3.25 µg/g DM [113]	2.0 µg/g [15] 0.46–0.50 µg/g [94] 4.69–16.62 µg/g DM [114]	Slightly strong (2 g/L—water) [92]		DT: 665 µmol/L (wine) [50]
Protocatechuic acid	99-50-3	154.1	67.6 µg/g [102]	0.33–0.41 µg/g [94] 8.28 µg/g DM [103] 0.00–2.40 µg/g [108]	28.3–48.0 µg/g DM [90]	D [71] 18.3 µg/g [111] 1.29–2.93 µg/g [88]	0.49–0.52 µg/g DM [94] 4.27 µg/g DM [103] 1.45 µg/g DM [113]	D [15] 2.06–2.21 µg/g [94] 2.77–19.82 µg/g DM [114]	Moderate (2 g/L—water) [92]		DT: 206 µmol/L (wine) [50]
p-Coumaric acid	7400-08-0	164.2	D [95] 31.3 µg/g [102] 0–180 µg/g DM [109]	D [95] 0.22 µg/g DM [103]	17.6–99.4 µg/g DM [90] 0–4.1 µg/g DM [96]	D [95,115] 25.8 µg/g [111] 0.95–1.86 µg/g [88]	D [95] 3.22–3.42 µg/g DM [94] 38.84 µg/g [112] 6.47 µg/g DM [113] 37.3 µg/g DM [116]	D [15,95] 0.38–0.41 µg/g [94] 0.54–1.10 µg/g DM [114]	Moderate (2 g/L—water) [92]		Sensory detection [92] DT: 139 µmol/L (wine) [50]
m-Coumaric acid	588-30-7	164.2				D [71]		D [95]	Moderate (2 g/L—water) [92]		DT: 292 µmol/L (wine)
Gallic acid	149-91-7	170.1	0–520 µg/g DM [109]	0.0–213.0 µg/g DM [97]	5 µg/g DM [13] 4.1–22.0 µg/g DM [90] 37.5–225.7 µg/g DM [96] 0.0–106.0 µg/g DM [97]	D [71] 26.9 µg/g [111]	2.54 µg/g DM [103] 100.0 µg/g DM [97]	0.016 µg/g [15]	Moderate (2 g/L—water) [92]		Sensory detection [92]
Caffeic acid	331-39-5	180.2		10.0–22.0 µg/g DM [13]	17.7–103.3 µg/g DM [90]			D [95] 20 µg/g DM [13] 0.091 µg/g [15]	Moderate (2 g/L—water) [92] DT: 0.11 mM [117] Based on a model *** [15]		Sensory detection [92] Based on a model *** [15]
**STILBENES**											
Resveratrol	501-36-0	228.2				D [98]			Bitter (wine) [101] DT: 47 mg/L (water) [67]	TAS2R14: AT = 16 µM; EC_50_ = 30.3 µM [20] TAS2R39: AT = 63 µM; EC_50_ = 109 µM [20]	Astringent (wine) [101]
**FLAVONOLS**											
Kaempferol	520-18-3	286.2	D [95] 0–90 µg/g DM [109]	D [95]	5.5–97.9 µg/g DM [90] 0–5.50 µg/g DM [96]	D [95]	D [95] 1.64 µg/g DM [103]	D [95]		TAS2R14: AT = 8 µM [20] TAS2R39: AT = 0.5 µM [20]	
Quercetin	117-39-5	302.2	D [95] 36.2 µg/g [102]	D [95] 1.91 µg/g [108]	7.0–104.9 µg/g DM [90] 0–14.5 µg/g DM [96]	D [95,98]	D [95]	D [95] 0–3 µg/g DM [97]		TAS2R14 (500 µM) [20] TAS2R39 (500 µM) [20]	
Myricetin	529-44-2	318.2			4.4–28.3 µg/g DM [90] 0–18.9 µg/g DM [96]					TAS2R14: AT = 250 µM [20] TAS2R39: AT = 1 µM [20]	
Quercetin-3-O-glucoside	482-35-9	464.4	D [105]	0.79 µg/g DM [104]			1.0 µg/g DM [103]	0.015 µg/g [15]			Based on a model *** [15]
**FLAVANOLS**											
(+)-Catechin	154-23-4	290.3	D [105] 0–160 µg/g DM [109]	32.15 µg/g DM [104] 142.58 µg/g [108] 0.0–23.0 µg/g DM [97] 132.38 µg/g [110]	4.7–92.4 µg/g DM [96] 0.0–26.0 µg/g DM [97]	D [98] 9.4 µg/g [111] 191–297 µg/g [88] 36.02 µg/g [112]	D [118] 0.1–0.3 µg/100 g DM [94] 0.53 µg/g DM [103] 0.77 µg/g DM [113]	D [15]	DT: 1000 µmol/L (water) [50] Weak (2 g/L—water) [92] Bitter (0.9 g/L—aqueous ethanol (1% *v*/*v*)) [106]	TAS2R14: AT = 500 µM [20] TAS2R39: AT = 250 µM [20]	DT: 410 µmol/L (water) [50,107] Astringent (0.9 g/L—aqueous ethanol (1% *v/v*)) [106]
(-)-Epicatechin	490-46-0	290.3	25.7 µg/g [102] 0–90 µg/g DM [109]			D [98] 98.25 µg/g [112]	70–97 µg/g DM [13] 4.17 µg/g DM [113]		DT: 930 µmol/L (water) [50] Moderate (2 g/L—water) [92] Bitter (0.9 g/L—aqueous ethanol (1% *v/v*)) [106]	TAS2R4: AT = 2000 µM; EC_50_ > 30151 µM [62] TAS2R5: AT = 1000 µM; EC_50_ = 3210 µM [62] TAS2R14: AT = 500 µM [20] TAS2R39: AT = 250–1000 µM; EC_50_ = 417.7–3800 µM [20,63,64]	DT: 930 µmol/L (water) [50,107] Astringent (0.9 g/L—aqueous ethanol (1% *v/v*)) [106]
(-)-Epigallocatechin	970-74-1	306.3				D [98]		0.00–1.61 µg/g DM [114]		TAS2R39: AT = 500 µM; EC_50_ = 395.5 µM [20,63]	DT: 520 µmol/L (water) [107]
(-)-Epicatechin gallate	1257-08-5	442.4				363 µg/g [111]				TAS2R14: AT = 125 µM; EC_50_ = 70 µM [20,64] TAS2R39: AT = 32 µM; EC_50_ = 21.3–151 µM [20,63,64]	DT: 260 µmol/L (water) [107]
(-)-Epigallocatechin gallate	989-51-5	458.4	0.1 µg/g [102]			18.3 µg/g [111]			DT: 380 µM [66]	TAS2R4 [66] TAS2R5: EC_50_ = 12.30 [66] TAS2R14: AT = 250 µM; EC_50_ = 34 µM [20,64] TAS2R30 [66] TAS2R39: AT = 32–100 µM; EC_50_ = 8.50–181.6 µM [20,63,64,66,119] TAS2R43: EC_50_ = 16.72 [66]	DT: 190 µmol/L (water) [107]
Theaflavin	4670-05-7	564.5				D [98]				TAS2R39: EC_50_ = 2.79 µM [64]	DT: 16 µmol/L (water) [107]
**FLAVONES**											
Chrysin	480-40-0	254.2	0–90 µg/g DM [109]			D [98,115]				TAS2R14: AT = 63 µM [20] TAS2R39: AT = 16 µM [20]	
7,4′-Dihydroxyflavone	2196-14-7	254.2				D [115]				TAS2R14: AT = 16 µM [20] TAS2R39: AT = 125 µM [20]	
Luteolin	491-70-3	286.2					D [118] 0.33 µg/g DM [113]			TAS2R14: AT = 2 µM; EC_50_ = 6.0 µM [20] TAS2R39: AT = 0.5 µM; EC_50_ = 7.3 µM [20]	
**FLAVANONES**											
Pinocembrin	480-39-7	256.2		1.26 µg/g [108]		D [98]				TAS2R14: AT = 8 µM; EC_50_ = 39.1 µM [20] TAS2R39: AT = 4 µM; EC_50_ = 48.9 µM [20]	
Naringenin	480-41-1	272.2					D [118]	0.082 µg/g [15]		TAS2R14: AT = 16 µM; EC_50_ = 36.2 µM [20] TAS2R39: AT = 8 µM; EC_50_ = 32.9 µM [20]	
**ISOFLAVONES**											
Daidzein	486-66-8	254.2		0.209 µg/g DM [120]	0.0–40.3 µg/g DM [90] 0.475 µg/g DM [120]	0.59 µg/g DM [120]	0.84 µg/g DM [120]	0.41 µg/g DM [120]	Slightly (1 µM) [80]	TAS2R14: AT = 500 µM [21] TAS2R39: AT = 500 µM [21]	Astringent (0.1–1 µM) [80]
Formomonetin	485-72-3	268.3				D [98]				TAS2R14: AT = 500 µM [21] TAS2R39: AT = 500 µM [21]	
Genistein	446-72-0	270.2		0.191 µg/g DM [120]	0.7–33.8 µg/g DM [90] 0.766 µg/g DM [120]	D [98] 0.74 µg/g DM [120]	0.139 µg/g DM [120]	0.144 µg/g DM [120]	Slightly (1.5 µM) [80]	TAS2R14: AT = 4 µM; EC_50_ = 28.9 µM [21] TAS2R39: AT = 8 µM; EC_50_ = 49.4 µM [21]	Weakly (10 µM) [80] Astringent (1.5 µM) [80]
**PROCYANIDINS**											
Procyanidin B1	20315-25-7	578.5		213.0 µg/g [108]		D [98]	D [118]		DT: 400 µM [50]	TAS2R5: EC_50_ = 119.34 µM [66] TAS2R7: EC_50_ = 123.95 µM [66]	DT: 240 µM [50]
Procyanidin B2g (3-O-gallate)	29106-49-8	578.5				D [98,111]	0.49 µg/g DM [103]			TAS2R5: EC_50_ = 6.29 µM [66] TAS2R39: EC_50_ = 9.11 µM [66]	
Procyanidin B4	29106-51-2	578.5		16.0 µg/g [108]		D [98]			Bitter (0.9 g/L—aqueous ethanol (1% *v/v*)) [106]	TAS2R5 [66]	Astringent (0.9 g/L—aqueous ethanol (1% *v/v*)) [106]
Procyanidin C2	-	866.8		42.4 µg/g [108]		D [98]			Bitter (0.9 g/L—aqueous ethanol (1% *v/v*)) [106]	TAS2R5: AT = 30.0 µM; EC_50_ = 35.6 µM [62]	Astringent (0.9 g/L—aqueous ethanol (1% *v/v*)) [106]

D: detected; DM: dry matter; EC_50_: half-maximum effective concentration of agonist required to achieve 50% of the maximum amplitude of receptor activation; DT: detection threshold; AT: activation threshold; TAS2R: human type 2 taste receptor. * CAS and molar mass (M) from the literature (NIST, 2022 and PubChem, 2022). ** Common beans include pulses from the specie *Phaseolus vulgaris*. *** Model based on the correlations of phytochemical compounds area determined by UHPLC-DAD-MS (ultrahigh-performance liquid chromatography–diode array detector–mass spectrometry) and sensory profiling [15].

- Flavones

Only three flavones have been detected in pulses, for which the activation of TAS2Rs has been studied. Chrysin has been identified in adzuki beans (0.00–0.09 g/kg) and faba beans [98,109,115], and activates TAS2R14 and TAS2R39 at concentrations of 63 µM and 16 µM, respectively [20]. 7,4′-Dihydroxyflavone, detected in faba beans, activates TAS2R14 at a lower threshold (16 µM) than chrysin, whereas it is higher for TAS2R39 (125 µM) [20,115]. Finally, luteolin exhibits very low AT for these two receptors in comparison with the previous flavones: 2 µM for TAS2R14 and 0.5 µM for TAS2R39 [20]. Pea flour contains 81.7 ng/g luteolin, but this compound does not contribute to bitterness and astringency according to the correlation model [15]. The concentrations of luteolin and caffeic acid are similar in pea flour, although only caffeic acid contributes to its bitterness and astringency [15]. One explanation is that the bitter receptor threshold activation of caffeic acid was lower than that of luteolin. However, this remains to be demonstrated.

- Flavanones

Roland et al. (2013) [20] have shown the activation of TAS2R14 and TAS2R39 by two flavanones, pinocembrin and naringenin. The TA and EC_50_ are similar for both molecules and bitter receptors [20]. Pinocembrin has been identified in common beans and faba beans, whereas lentils contain naringenin [98,108,118].

- Isoflavones

Three isoflavones, daidzein, formononetin, and genistein, have been identified in beans, chickpeas, faba beans, lentils, and peas. The extracts of daidzein and genistein from soybeans are described as slightly bitter and astringent by a trained panel [80]. These isoflavones are involved in the bitter taste and astringency of soybeans due to their very high content compared to other legumes (Table 5) [18,19]. Genistein (extracted from soybeans) has AT for TAS2R14 and TAS2R39 at concentrations of 4 and 8 µM, respectively [21], and these ATs are similar to naringenin (flavanone) [20]. These low threshold values could explain the important role of genistein in the perception of soybean bitterness. However, the relationship between the AT (receptor level) and DT (sensory level) has never been demonstrated for this compound. Moreover, daidzein and formononetin (also extracted from soybeans) also activate these two receptors at a higher concentration (500 µM) [21]. The number and positions of hydroxyl groups should be an important parameter for TAS2R activation [20,66]. Indeed, genistein exhibits three OH groups whereas formononetin and daidzein have one and two hydroxyl groups. However, chickpeas, soybeans, and peas are more concentrated in daidzein than in genistein. These two isoflavones could be equally involved in the bitter sensation of these pulses (balance between concentration and AT). In addition, malonyl-β-glucosides such as malonyl daidzein, malonyl glycitin and malonyl genistein have been identified in soybean flakes [81]. These are derived from the malonylation of β-glucosides [121]. These malonyl-β-glucosides should contribute as much to soybean bitterness and astringency as DDMP saponin and more than the other saponins and isoflavones [81]. Currently, none of these malonyl-β-glucosides have been identified in beans, chickpeas, faba beans, lentils and peas. Finally, heat treatment reduces the isoflavone content, whereas germination increases it [120,122].

#### 3.2.4. Condensed Tannins

Condensed tannins are oligomers or polymers composed of derivates from (+)-catechin and its isomers. Unlike hydrolysable tannins, they are resistant to hydrolysis and are degraded using chemical treatments [123]. Prodelphinidins and procyanidins have been identified in pulses. Hulls contain higher concentrations of these condensed tannins than cotyledons [90,91,124]. These compounds may be responsible for bitterness and astringency in grapes and wines [125]. In lupin, condensed tannins may be more involved in bitterness than flavanols and alkaloids [16]. Moreover, the evaluation of the bitter and astringent intensities of low- and high-tannin faba beans would have made it possible to verify their involvement [89].

Procyanidins B4 and C2 are described as astringent and bitter at a concentration of 0.9 g/L in aqueous ethanol [106]. Dimers and trimers of procyanidins are more astringent than monomers ((+)-catechin and (-)-epicatechin) [106]. The astringent DT of (-)-epicatechin is five times higher than that of procyanidin B2 [50]. Hufnagel and Hofmann (2008) suggested that the more polymerised the molecules are, the more bitter they are, as shown by the ranking obtained according to the intensity of bitterness perceived in wines: procyanidin B1 and C1 > procyanidin B2 > procyanidin B3 > (-)-epicatechin > (+)-catechin [50]. Indeed, a similar ranking of TAS2R5 receptor DTs has been established [62]. Conversely, Peleg et al. (1999) demonstrated using sensory analysis that the more polymerised the molecules are, the less bitter they are. In wine, (-)-epicatechin is more bitter than (+)-catechin, which is more bitter than procyanidin trimers [106]. These contradictory results can be explained by the presence of ethanol in the wines, which increases the intensity of the bitter perception in the mouth. Indeed, Fischer and Noble (1994) have shown that an increase of 3% (*v/v*) ethanol in wine is equivalent to an increase in bitterness (+50%) caused by the addition of 1400 mg/L catechin [126]. However, it is not possible to verify these results with bitter receptors in vitro in the presence of ethanol, as this molecule is 1% toxic to cells. Some sensorial results are therefore consistent with those obtained via cell tests: the degree of polymerisation of these phenolic compounds should increase the intensity of bitterness. (+)-Epicatechin activates TAS2R4, TAS2R5 and TAS2R39 from a concentration above 1000 µM, while procyanidin C2 (trimer) activates TAS2R5 from 30 µM [62]. Roland et al. (2011) suggest that a molecule with many hydroxyl groups could have a better affinity for TAS2R5 [21]. Indeed, dimers (procyanidins B) and trimers (procyanidins C) have more OH groups than monomers. The ability of seven procyanidins (five dimers and two trimers also identified in pulses) to activate the 25 TAS2Rs has been tested (Table 6) [62,66]. Only TAS2R5, TAS2R7 and TAS2R39 are activated by at least one procyanidin. Procyanidins B2, B3, and C1 did not activate the 25 TAS2Rs at the tested concentrations. TAS2R7 is only activated by procyanidin B1, and TAS2R39 is activated by procyanidin B2g. In addition, TAS2R5 is activated by procyanidins B1, B2g, B4 and C2. Procyanidin B2g exhibits the lowest EC_50_ followed by procyanidins C2 and then B1. However, the EC_50_ of dimer B4 has not been determined due to the observation of unspecific responses in the control condition (mock) [62,66]. These results suggest a role for condensed tannins, especially procyanidins, in the bitterness and astringency of pulses. However, it would be interesting to investigate the potential bitter taste and astringency of prodelphinidins.

### 3.3. Alkaloids

Some alkaloids contribute to the bitterness of food products such as caffeine [6]. Approximatively sixteen alkaloids have been detected in different lupin varieties and could be partially responsible for their bitterness. They are distributed in the quinolizidine, indole, and piperidine classes [16,127,128]. For example, lupanine is the most abundant alkaloid in white and narrow-leafed lupins and sparteine in yellow lupins [128]. However, quinolizidine alkaloids are considered human antinutritional factors due to neurological, cardiovascular, and gastrointestinal disturbances [128,129]. Lupins are classified into two varieties: the “bitter” and the “sweet” which differ in their alkaloid content [16,130]. DuPont et al. established the relationship between the bitter intensity of milled lupins and their alkaloid content [16]. The bitter mean scores of the “bitter” varieties are higher than those of the “sweet” ones (7.8 and 2.0 over 10, respectively). The “sweet” varieties exhibited 0.1 mg/g DM of mean alkaloids compared to 15.0 mg/g DM for the “bitter” varieties. Concerning the “bitter” varieties, the lupin evaluated as the least bitter contains 4.8 mg/g (dry matter) of alkaloids, including lupinine and gramine, whereas the one with the highest bitter intensity contains 26.9 mg/g (dry matter) composed of sparteine, lupanine and 13-hydroxylupanine. This study has highlighted the role of alkaloids in lupin bitterness; however, the authors suggest that intense bitterness in “bitter” varieties could also be attributed to the presence of tannins [16]. Moreover, treatments to eliminate alkaloids in lupin are called “debittering treatments” [127].

Faba bean is another pulse containing two alkaloids, vicine and convicine [131,132]. These molecules are pyrimidine glucosides and cause favism in people who express a genetically inherited glucose-6-phosphate dehydrogenase (G6PD) deficiency; they are considered antinutritional factors [89,133,134]. Such as lupins, there are new cultivars of faba bean breeding lines with a significantly lower amount of alkaloids; the reduced level of vicine and convicine can considerably vary among cultivars [89,133]. However, the sensorial aspect of these molecules has never been studied. It would be interesting to compare the bitter intensity of the high- and low-vicine/convicine cultivars. A study correlated the flavour-related components and the sensorial attributes of faba bean flour, concentrate and isolate using partial least squares (PLS) regression. Bitterness is related to vicine and convicine, although other compounds, including free phenolic compounds and amino acids (phenylalanine, tryptophan, and histidine), also contribute [57].

Thus, the main disadvantages of alkaloids are their antinutritional effect and their potential contribution to bitterness of lupins and faba beans. However, the main advantage of these secondary metabolites is their involvement in the plant mechanism, which limits herbivory attacks and ensures harvest yields [12,135]. For example, the low vicine and convicine faba bean genotypes are more sensitive to bruchid attack [89]. These compounds are beneficial for the plant in the field and can be eliminated after harvesting by many strategies including cooking, soaking, germination, and fermentation [127,134,136,137]

## 4. Conclusions

Consumers and food industries generate constant demand for plant-based products, but there remains a strong need to improve their flavour. The volatile compounds of many pulses and their impact on off-notes have been relatively well-studied. However, this review concludes that there is a gap in the knowledge regarding non-volatile compounds causing bitterness and astringency in pulses. It is therefore important to identify the molecules involved in plant-based off-flavours to increase consumer acceptability.

This review compiles the different non-volatile compounds that contribute to the bitterness and astringency of pulses and potential compounds identified in pulses that are described as bitter and astringent in other food products. All the molecules listed in this review originate from a secondary metabolism and contribute to pulse defence. They are more cultivar- and pulse-dependent, and their content varies owing to differences in environmental, storage and transformation conditions. Saponins may be the main compounds responsible for bitterness and astringency in peas and soybeans. Due to the high saponin content in chickpeas, this family of compounds could also be responsible for off-flavours. However, lupins and faba beans have lower saponin contents and differ from other legumes in the presence of alkaloids which may also contribute to bitterness. According to phenolic compounds, isoflavones are responsible for bitterness and astringency in soybeans but are probably not involved in other pulse off-flavours due to their very low content. Moreover, the number of phenolic compounds identified in pulses that exhibit bitter and/or astringent characteristics is very large; however, their role in pulse off-flavours needs to be highlighted, although some predictions suggest the possibility. It would be interesting to continue the study of these compounds through both sensory analysis and in vitro tests. Cellular in vitro tests provide information on activated TAS2R and make it possible to target the most impactful compounds on off-flavours. Moreover, the activation of the same TAS2R by different molecules and the activation of multiple TAS2Rs by a single molecule may lead to an increase in perceived bitterness. It is also interesting to compare the molecule content of pulses and their TAS2R AT to verify their involvement in off-flavours.

This review is probably not exhaustive and might be completed in the coming years. Other compounds, particularly many phenolic compounds, have been identified in legumes; however, their sensory impact (bitterness and astringency) on food products has never been studied, which could suggest new potential compounds. Indeed, studies often focus on the identification of molecules alone or the overall bitterness and/or astringency of plant-based products without linking to their content. Moreover, recent studies have suggested the role of other compounds, including lipids, lipid oxidation products, peptides and free amino acids, in the negative perception of pulses [9,22,138,139].

Finally, precise identification of bitter and astringent compounds in pulses could allow the determination of their main origins and the proposal of strategies to reduce their perception. Some approaches have been identified for improving the flavour of pulses, such as limiting the production of these unwanted compounds, removing them or masking their perception. One approach consists of selecting cultivars with low saponin content (peas) or with low alkaloid and tannin content (faba beans and lupins). However, this strategy decreases plant resistance and may raise other problems such as low production yields. The content of these compounds may vary during seed transformation, showing that a compromise must be considered. For example, germination reduces the alkaloid seed content while enriching it with isoflavones and saponins. The fermentation of plant-based products decreases the content of these unwanted compounds but promotes the formation of new bitter compounds such as peptides and free amino acids [140]. Another approach consists of using perceptual interactions to limit intensity of bitterness and astringency, in particular using odour-taste interactions [141,142].

## Figures and Tables

**Table 1:** Comparison between human (in vitro) and cellular (in vivo) bitter characteristics for different compounds.

Bitter Compounds	Human Bitter Characteristics		Cellular Bitter Characteristics			Ratio (Human/Cellular)		References
	DT (µM)	EC_50_ (µM)	TAS2R	AT (µM)	EC_50_ (µM)	Threshold	EC_50_	
phenyl-β-D-glucopyranose	100	700	TAS2R16	70	1100	1.4	0.6	[53]
salicilin	200	1100	TAS2R16	70	1400	2.9	0.8	[53]
helicin	400	2200	TAS2R16	300	2300	1.3	1.0	[53]
arbutin	900	5400	TAS2R16	500	5800	1.8	0.9	[53]
2-nitrophenyl-β-D-glucopyranose	ND	-	TAS2R16	1500	ND	NC	NC	[53]
naphthyl-β-D-glucopyranose	200	1400	TAS2R16	400	1000	0.5	1.4	[53]
methyl-β-D-glucopyranose	32,000	320,000	TAS2R16	15,000	ND	2.1	NC	[53]
amygdalin	ND	-	TAS2R16	2300	20,000	NC	NC	[53]
esculin	4000	ND	TAS2R16	4000	ND	1	NC	[53]
phenyl-β-D-galactopyranose	40,000	ND	TAS2R16	ND	-	NC	NC	[53]
phenyl-α-D-glucopyranose	9000	50,000	TAS2R16	ND	-	NC	NC	[53]
phenylthiocarbamide	PAV: 3.28 AVI: 1360	-	TAS2R38-PAV TASR238-AVI	0.02 ND	1.1 -	NC-164	NC	[40]
propylthiouracil	PAV: 10.7 AVI: 413	-	TAS2R38-PAV TASR238-AVI	0.06 ND	2.1 -	NC-178.3	NC	[40]
*trans*-isocohumulone	19	300	TAS2R1 TAS2R14	1 1	10.6 14.5	19	20.7–28.3	[25]
*trans*-isohumulone	20	200	TAS2R1	0.3 1	9.0 11.2	20–66.7	17.8–22.2	[25]
*trans*-isoadhumulone	13	130	TAS2R14	0.3 1	6.7 9.0	13–43.3	14.4–19.4	[25]
*cis*-isocohumulone	7	180	TAS2R1	1 1	7.4 9.4	7	19.1–24.3	[25]
cis-isohumulone	10	110	TAS2R14	0.3 0.3	3.3 2.6	33.3	33.3– 42.3	[25]
cis-isoadhumulone	8	100	TAS2R1	0.3 1	2.5 2.8	8–26.7	35.7–40	[25]
cohumulone	17	>500	TAS2R1 TAS2R40	0.03 0.003	0.2 0.04	566.7–5667.7	NC	[25]
humulone	21	ND	TAS2R1 TAS2R40	0.1 0.1	1.4 0.4	210	NC	[25]
adhumulone	21	ND	TAS2R1 TAS2R40	0.1 0.03	0.7 0.2	210–700	NC	[25]
colupulone	39	>500	TAS2R1 TAS2R40	0.1 0.03	0.7 0.2	390–1300	NC	[25]
lupulone	35	ND	TAS2R1	0.1 3	3.0 1.3	11.7–350	NC	[25]
adlupulone	37	ND	TAS2R14	1 3	2.2 4.1	12.3–37	NC	[25]
isoxanthohumol	16	>500	TAS2R1 TAS2R14 TAS2R40	3 3 10	ND ND ND	1.6–5.3	NC	[25]
xanthohumol	10	140	TAS2R1 TAS2R14 TAS2R40	1 3 3	ND ND ND	3.3–10	NC	[25]
8-prenylnaringenin	8	ND	TAS2R14	0.3	1.5	26.7	NC	[25]
vitamin B1	1100	-	TAS2R1	100	-	11.0	-	[51]
vitamin B2	650	-	-	-	-	-	-	[51]
vitamin B3	5500	-	-	-	-	-	-	[51]
vitamin B6	5200	-	TAS2R7 TAS2R14	1000 1000	25,520 10,520	5.2	NC	[51]
vitamin A	ND	-	TAS2R38	50	290	NC	NC	[51]
vitamin D	ND	-	TAS2R10	50	250	NC	NC	[51]
resveratrol	206	-	TAS2R14 TAS2R39	16 63	30.3 109	3.3–12.9	NC	[20,67]
(+)-catechin	1000	-	TAS2R14 TAS2R39	500 250	ND	2–4	NC	[20,50]
(-)-epicatechin	930	-	TAS2R4 TAS2R5 TAS2R14 TAS2R39	2000 1000 500 250–1000	>30,151 3210 500 418–3800	0.5–3.7	NC	[20,50,62,63,64]
(-)-epigallocatechin gallate	380	-	TAS2R14 TAS2R39	250 32–100	-	1.5–11.9	-	[20,63,64,66]

The first part presents the in vivo and in vitro results from the same study, while the second part (separated by a double line) is from several references. DT: detection threshold; AT: activation threshold; EC_50_: half-maximum effective concentration corresponding to the concentration of compound required to achieve 50% of the bitter intensity (in vivo)/to achieve 50% of the maximum amplitude of receptor activation (in vitro); PAV: proline-alanine-valine; AVI: alanine–valine–isoleucine; ND: not determined; NC: not calculable.

**Table 2:** Concentration (mg/g DM) of phenolic compounds, flavonoids and tannins in different seed parts of pulses (chickpeas, faba beans and lentils).

Pulses	Concentration (mg/g DM)												Reference
	Whole Seed			Cotyledon			Hull			Embryonic Axe			
	PC	F	T	PC	F	T	PC	F	T	PC	F	T	
Chickpeas	-	-	-	15.2	7.5	5.2	75.9	12.6	32.4	46.1	9.3	11.4	[90]
Faba beans	39.69	-	6.85	39.17	-	7.22	22.30	-	16.23	-	-	-	[91]
Lentils	6.30	-	1.27	4.27	-	0.40	57.19	-	46.27	-	-	-	[91]

DM: dry matter; PC: phenolic compounds; F: flavonoids; T: tannins.

**Table 4:** TAS2Rs activated by some flavanols identified in pulses (adzuki beans, beans *, chickpeas, faba beans, lentils and peas).

Flavanols	TAS2R4	TAS2R5	TAS2R14	TAS2R30	TAS2R39	TAS2R43	Reference
(+)-catechin			+ (500; ND)		+ (250; ND)		[20]
(-)-epicatechin	+ (2000; ≥30,151)	+ (1000; 3210)	+ (500; ND) − (≤100)	− (≤100)	+ (250; ND) + (1000; 3800) + (ND; 417.7)	− (≤100)	[20] [62] [63]
(-)-epigallocatechin			− (≤500)		+ (500; ND) + (ND; 395.5)		[20] [63]
(-)-epicatechin gallate			+ (125; ND) + (ND; 70)		+ (32; 151) + (ND; 21.3) + (ND; 88.2)		[20] [64] [63]
(-)-epigallocatechin gallate	+ (≤100; ND)	+ (≤100; 12.30)	+ (250; ND) − (≤100) + (ND; 34)	+ (≤100; ND)	+ (32; 161) + (≤100; 8.50) + (ND; 112) + (ND; 181.6)	+ (≤100; 16.72)	[20] [66] [64] [63]
Theaflavin			− (ND)		+ (ND; 2.79)		[64]

ND: not determined. “+” indicated receptor activation followed by activation threshold (µM) and EC_50_ (half-maximum Effective Concentration) (µM); “−“ indicated a lack of activation followed by the higher concentration tested (µM). * Beans include pulses from the specie *Phaseolus vulgaris*.

**Table 5:** Concentration (µg/kg DM) of genistein and daidzein in different legumes [120].

Legumes	Genistein (µg/kg DM)	Daidzein (µg/kg DM)
broad beans (raw)	74	59
chickpeas (dried, raw)	475	766
lentils (dried, raw)	139	84
red kidney beans (raw)	191	209
soybeans (dried, raw)	583.10^3^	838.10^3^
peas (dried, raw)	41	144

DM: dry matter.

**Table 6:** hTAS2Rs activated by dimer and trimer condensed tannins identified in pulses (beans *, faba beans and lentils).

	Procyanidins	TAS2R5	TAS2R7	TAS2R39	Reference
**dimers**	B1	+ (≤67; 119.34)	+ (≤67; 123.95)	− (≤67)	[66]
	B2	− (≤67)	− (≤67)	− (≤67)	[66]
	B2g (3-O-gallate)	+ (≤100; 6.29)	− (≤100)	+ (≤100; 9.11)	[66]
	B3	− (≤67)	− (≤67)	− (≤67)	[66]
	B4	+ (≤133; ND)	− (≤133)	− (≤133)	[66]
**trimers**	C1	− (≤150)	− (≤150)	− (≤150)	[66]
	C2	+ (30.0; 35.6)	− (≤300)	− (≤300)	[62]

ND: not determined. “+” indicated receptor activation followed by activation threshold (µM) and EC_50_ (half-maximum effective concentration to achieve 50% of the maximum amplitude of receptor activation) (µM); “−“ indicated a lack of activation followed by the higher concentration tested (µM). * Beans include pulses from the specie *Phaseolus vulgaris*.

## Data Availability

Not applicable.
